# Changes in Mental Well-Being, Lifestyle Behaviors, and Medication Use Before and During Medical School

**DOI:** 10.7759/cureus.106271

**Published:** 2026-04-01

**Authors:** Amira Katrib, Kelson Knighton, Asis A Babun, Thomas Bigham

**Affiliations:** 1 Military Medicine, Rocky Vista University College of Osteopathic Medicine in Southern Utah, Ivins, USA; 2 Biomedical Sciences, Rocky Vista University College of Osteopathic Medicine in Southern Utah, Ivins, USA; 3 Family Medicine, Rocky Vista University College of Osteopathic Medicine in Southern Utah, Ivins, USA; 4 Primary Care Medicine, Rocky Vista University College of Osteopathic Medicine in Southern Utah, Ivins, USA

**Keywords:** medical students, mental health, physical activity, psychiatric medication, stress

## Abstract

Background: Medical training is commonly associated with increased psychological stress, yet changes in mental health from before to during medical school are not consistently evaluated using within-participant comparisons. This study aimed to assess whether mental health indicators and related behaviors worsen during medical school compared to premedical school status.

Methods: We conducted a cross-sectional, anonymous survey of current U.S. medical students and recent graduates between April 2025 and January 2026. Using a self-developed questionnaire, we assessed recalled mental health status before medical school and current experiences during medical school via adaptations of the Patient Health Questionnaire-9 (PHQ-9), General Anxiety Disorder-7 (GAD-7), and Social Functioning Scale (SFS). Additional self-developed items evaluated medication use, caffeine and nicotine use, physical activity, and perceived stressors. Paired ordinal outcomes were analyzed using Wilcoxon signed rank tests, and categorical variables were analyzed using McNemar tests.

Results: A total of 118 respondents completed all survey items. Participants reported significant increases in frequent mental and physical exhaustion (+62, 52.5%, *P *< 0.001) and frequent self-doubt (+33, 28.0%, *P *< 0.001) during medical school compared to before enrollment. Physical activity declined (*P *< 0.001), with a decrease of 31 (26.3%) in frequent exercise and a 3.4-fold increase in respondents reporting no regular exercise. Use of prescribed antidepressants and attention-deficit/hyperactivity disorder (ADHD) medications did not significantly differ before versus during medical school (*P *= 0.68 and *P *= 0.38, respectively). Caffeine use was common (103, 87.3%), and use during medical school increased for 68 (57.6%) of students, while nicotine use was uncommon (105, 89% never used). Academic workload, lack of free time, and difficulty balancing personal well-being were the most frequently reported stressors.

Conclusions: Medical school was associated with worsening self-reported mental health symptoms and declines in health promoting behaviors, without a corresponding increase in prescribed psychiatric medication use. These findings suggest that psychological distress during medical training may not be adequately addressed through formal treatment pathways, highlighting the need for preventive and supportive institutional interventions.

## Introduction

Mental well-being is a holistic state of psychological and emotional health that enables individuals to cope with normal life demands. However, medical education often disrupts this baseline. Previous surveys have identified that there is a high prevalence of burnout (an occupational syndrome involving emotional exhaustion and depersonalization), anxiety (excessive and persistent worry), and depression (a mood disorder characterized by prolonged sadness and loss of interest) among medical students [1-5]. A 2016 meta-analysis by Rotenstein et al. estimated the prevalence of depression or depressive symptoms at 27.2% among medical students [4]. Beyond the intrinsic value of mental well-being, psychological distress is closely associated with poorer academic performance [6], though this relationship may not be strictly causal [7]. The high prevalence of poor mental well-being represents a significant burden upon students. 

In light of this significant burden, it is important to understand how students cope with the stress they are placed under. The same 2016 meta-analysis showed that in the students who screened positive for depression, only 15.7% sought medical attention [4]. However, students may seek help through other avenues. A survey by Ley et al. found that up to 48% of students sought help for mental health concerns at some time during their time in medical school [2]. A few studies have investigated how students use psychotropic medications to help with their mental health. A survey of Brazilian medical students showed 30.4% used at least one psychiatric medication [8]. Another survey from Saudi Arabian medical students reported that 43.4% of students were using medications for depression, anxiety, or both [9]. To our knowledge, no previous study has investigated the prevalence of psychiatric medication use in medical students from the United States of America. 

These academic pressures frequently manifest as mental and physical exhaustion, which is defined as a severe depletion of cognitive and energetic resources [2,3]. Students also commonly experience self-doubt, characterized by pervasive feelings of inadequacy regarding their clinical or academic abilities [6]. Furthermore, a student's overall mental well-being, meaning their baseline psychological health and resilience, is heavily influenced by daily lifestyle behaviors [7]. These behaviors include routine, modifiable habits, such as physical activity, social engagement, and substance use, which often fluctuate in response to stress [2].

We sought to understand the current burden of stress and anxiety among medical students and how that population uses substances, prescription and over-the-counter, to manage their symptoms. To investigate this, we asked students about current symptoms and how their use of various substances changed after beginning medical school. Specifically, the objectives of this study were to (1) quantify within-subject changes in mental well-being by comparing retrospective self-reported frequencies of exhaustion, self-doubt, and stress before medical school to current frequencies during medical school; (2) evaluate the impact of medical training on health-promoting lifestyle behaviors by measuring alterations in the frequency of physical exercise and social engagement; and (3) determine if the transition into medical school is associated with a change in the utilization of prescribed psychiatric medications (antidepressants and attention-deficit/hyperactivity disorder (ADHD) medications) and non-prescription substances (caffeine and nicotine).

## Materials and methods

We conducted a quantitative, cross-sectional study between April 2025 and January 2026 through an anonymous online survey. The study protocol was reviewed and approved by the Institutional Review Board (IRB) at Rocky Vista University (Approval Number: 2024-257). All participants provided explicit informed consent digitally before accessing the questionnaire. To ensure participant confidentiality, no personally identifiable information was collected. Participants were recruited using convenience sampling. The inclusion criteria were current osteopathic medical students and recent graduates at Rocky Vista University College of Osteopathic Medicine. Participants were excluded from the final analysis if they failed to complete all required survey questions. Responses were collected using Qualtrics (Qualtrics, Provo, UT).

Survey items assessed mental health status, health-promoting behaviors, and medication use before and during medical school. To assess current psychological distress, we utilized a combined question block adapted from the Patient Health Questionnaire-9 (PHQ-9) [10], General Anxiety Disorder-7 (GAD-7) [11], and concepts from the Social Functioning Scale (SFS) [12]. Rather than administering these as standardized, clinical instruments with a strict two-week recall period, we modified the wording to evaluate general symptom frequency. No formal permissions were required for the adaptations to these surveys. The original PHQ-9 contains 9 items, scored from 0 to 3, to calculate depression severity; the GAD-7 contains 7 items, scored from 0 to 3, to calculate anxiety severity; and the SFS contains 79 items evaluating various domains of social adjustment. Rather than administering these as standardized clinical instruments to generate cumulative diagnostic scores, we extracted and modified core concepts to evaluate general symptom frequency. Our adapted instrument consisted of five stress/anxiety items and five social functioning items. Respondents rated these specific symptoms on individual four-point ordinal scales (e.g., *Never* to *Almost every day*). Because the items were analyzed individually to track symptom frequency, cumulative scoring criteria and diagnostic interpretation ranges were not utilized. This custom questionnaire was developed collaboratively by the study investigators; formal expert evaluation, pilot testing, and psychometric validation of the adapted instrument were not conducted before deployment. Additional sections evaluated changes in the use of psychiatric medications, caffeine, nicotine, and physical activity, comparing recalled pre-medical school usage to current usage. The full survey is available in the Appendix.

**Figure 1:**
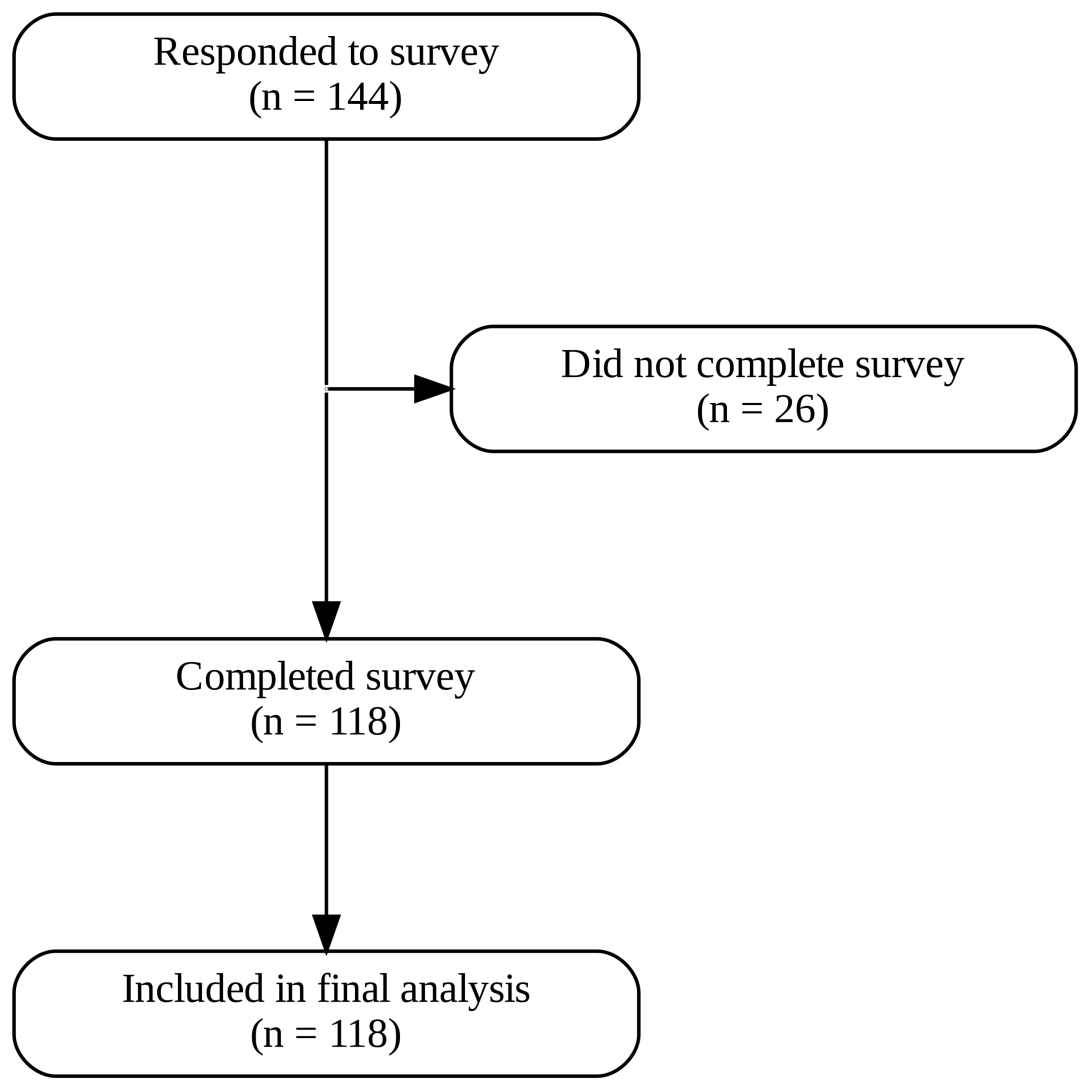
CONSORT diagram of survey participants Image credit: Kelson Knighton.

Data were analyzed using Python 3.14.3. Data manipulation and all statistical testing were executed utilizing the NumPy, pandas, and SciPy libraries. To determine the appropriate statistical approach, the assumption of normality was formally assessed using the Shapiro-Wilk test, which confirmed non-normal distributions across the tested variables. Consequently, non-parametric methods were utilized: paired ordinal outcomes were analyzed using Wilcoxon signed-rank tests, and categorical variables were analyzed using McNemar's test. All data visualizations were generated utilizing the matplotlib and seaborn libraries. Statistical significance for all tests was defined as a two-tailed *P*-value of < 0.05. Finally, a formal a priori sample size calculation was not performed; rather, a convenience sampling approach was utilized to capture representative responses during the open survey window. Effect sizes were calculated using point-biserial correlation. A total of 144 people responded to the survey, of which 118 (82%) completed all questions and were included in the final analysis (Figure 1).

## Results

The study sample comprised a total of 118 medical students, with a slight majority identifying as female, 67 (56.8%), compared to male 51 (43.2%). The age distribution was heavily concentrated in the 25-29 years old age bracket, which represented 73 (61.9%) of the total population, while younger students (18-24 years) accounted for 28 (23.7%). In terms of academic progression, the sample showed a relatively even distribution across the first three years of medical school, with Year I (40, 33.9%), Year II (35, 29.7%), and Year III (35, 29.7%), making up the bulk of the respondents. Participation was much lower among Year IV and recently graduated students; combined, they made up only 8 (6.8%) of the cohort (Table 1).

**Table 1:** Baseline demographic breakdown of survey respondents (n = 118). Data reflect the number and percentage of participating medical students across gender categories, age brackets, and academic progression.

Characteristics	Frequency (*n*)	Percentage (%)
Total participants	118	100.0
Gender		
Female	67	56.8
Male	51	43.2
Age group (years)		
18-24	28	23.7
25-29	73	61.9
30-34	9	7.6
35+	8	6.8
Year in Medical School		
Year I	40	33.9
Year II	35	29.7
Year III	35	29.7
Year IV	6	5.1
Graduate	2	1.7

**Figure 2:**
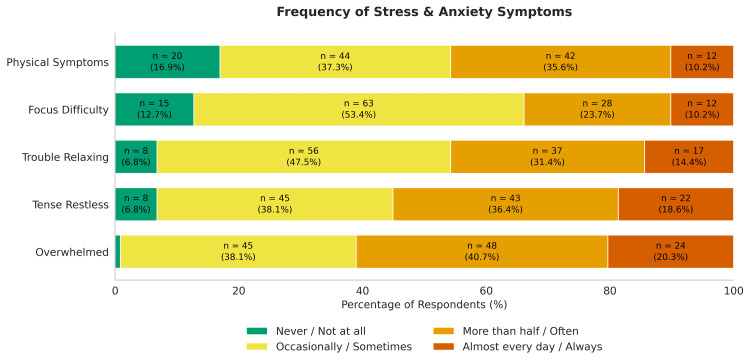
Distribution of self-reported stress and anxiety symptoms among medical students (n = 118). Participants rated the frequency of feeling overwhelmed, tense/restless, unable to relax, unable to focus, or experiencing physical symptoms on a four-point scale ranging from *Never*/*Not at all *to *Almost every day*/*Always*.

A large number of students reported experiencing negative sensations associated with stress and anxiety (Figure 2). The most common of these was feeling overwhelmed, with 72 (61%) reporting feeling overwhelmed more than half the time or almost every day (Table 2). Physical symptoms of stress (such as headaches, muscle tension, or stomach discomfort) were relatively less common. 

**Table 2:** Frequency of stress and anxiety symptoms among medical students. Data represent the distribution of self-reported stress and anxiety symptoms (*n* = 118). Participants rated the frequency of each symptom on a four-point ordinal scale ranging from *Never*/*Not at all *to *Almost every day*/*Always*. Values are presented as frequency (*n*) and percentage (%).

Variable	n	%
Feeling overwhelmed by responsibilities		
Almost every day	24	20.3
More than half the time	48	40.7
Occasionally	45	38.1
Never	1	0.8
Feeling tense, restless, or on edge		
Almost always	22	18.6
Often	43	36.4
Sometimes	45	38.1
Not at all	8	6.8
Trouble relaxing or calming thoughts		
Almost every day	17	14.4
More than half the time	37	31.4
Occasionally	56	47.5
Never	8	6.8
Difficulty focusing due to stress/anxiety		
Almost every day	12	10.2
More than half the time	28	23.7
Occasionally	63	53.4
Never	15	12.7
Physical symptoms of stress		
Almost always	12	10.2
Often	42	35.6
Sometimes	44	37.3
Not at all	20	16.9

Although a majority of students reported engaging in social interactions at least a few times per week (85, 72.1%) and felt comfortable in social settings (72, 61.0%), stress and anxiety took a notable toll on their interpersonal lives (Table 3). Specifically, 69 (58.5%) of respondents indicated that stress had a negative impact on their relationships. Furthermore, nearly one-third of students (39, 33.1%) reported avoiding social situations more than half the time or almost every day due to feeling overwhelmed, anxious, or exhausted. Participation in personal interests was also limited, with only 23 (19.5%) of students reporting regular engagement in hobbies.

**Table 3:** Social well-being and interactions among medical students.

Variable	Frequency (*n*)	Percentage (%)
Frequency of social interaction		
Daily	39	33.1
A few times per week	46	39.0
Occasionally	24	20.3
Rarely or never	9	7.6
Comfort in social situations		
Extremely comfortable	22	18.6
Moderately comfortable	34	28.8
Slightly comfortable	16	13.6
Neither comfortable nor uncomfortable	16	13.6
Slightly uncomfortable	24	20.3
Moderately uncomfortable	4	3.4
Extremely uncomfortable	2	1.7
Impact of stress/anxiety on social relationships		
Extremely positive	1	0.8
Moderately positive	3	2.5
Slightly positive	12	10.2
Neither positive nor negative	33	28.0
Slightly negative	44	37.3
Moderately negative	21	17.8
Extremely negative	4	3.4
Avoiding social situations		
Almost every day	8	6.8
More than half the time	31	26.3
Occasionally	63	53.4
Never	16	13.6
Participation in hobbies/activities		
Yes, regularly	23	19.5
Occasionally	62	52.5
Rarely	31	26.3
Not at all	2	1.7

**Figure 3:**
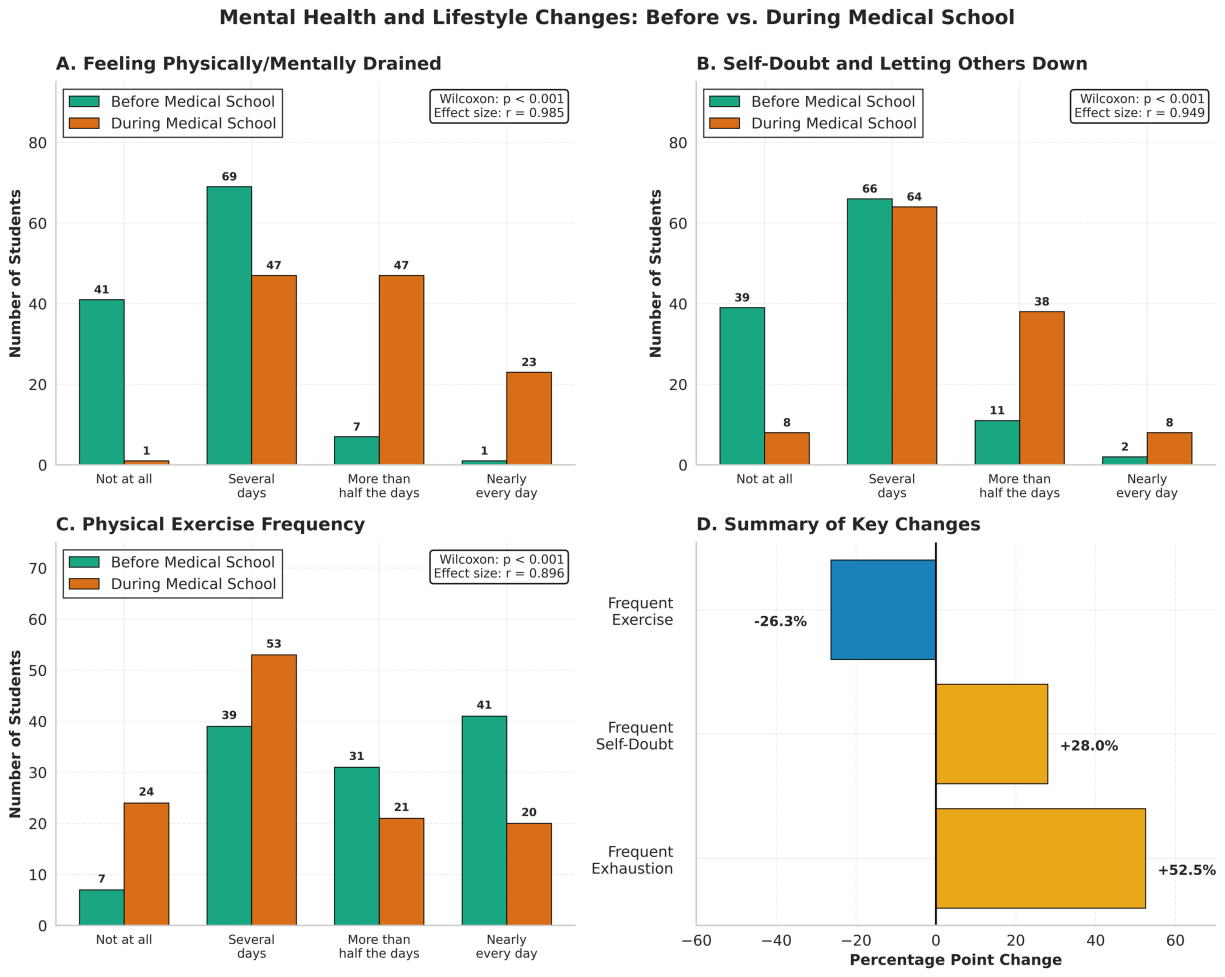
Mental health and lifestyle changes: before vs. during medical school. Histograms of survey responses are shown in panels (A)-(C). Significance for the trend in each panel was calculated using the Wilcoxon signed-rank test. Effect sizes were calculated using point-biserial correlation. Answers of *More than half the days* and *Nearly every day *were combined into the *frequent *category for panel (D).

Participants were asked questions about their current mental health as well as to recall how they felt before entering medical school (Figure 3). Participants reported rarely feeling mentally or physically drained before medical school but commonly felt drained during medical school (Figure 3A). Feelings of self-doubt also increased after enrollment (Figure 3B). The frequency of physical exercise also decreased significantly. The number of participants who reported never exercising increased by 3.4x, rising from 7 (5.9%) students before enrollment to 24 (20.3%) students during medical school (Figure 3C). Figure 3D summarizes how the number of people reporting that they exercised *More than half the days* or *Nearly every day* decreased by 31 (26.3%), while frequent feelings of self-doubt and exhaustion increased by 33 (28%) and 62 (52.5%), respectively. Responses are also available in a tabular format in Table 4.

**Table 4:** Frequency of mental health symptoms and lifestyle behaviors before and during medical school. Data are presented as raw frequency counts (*n*), representing a total of 118 participants, and as percentages of the total. Respondents rated the frequency of each parameter on a four-point ordinal scale.

Variable	Timepoint	Not at all	Several days	More than half	Nearly every day	*P*-value	Effect size (*r*)
Feeling drained	Before	41 (34.7%)	69 (58.5%)	7 (5.9%)	1 (0.8%)		
	During	1 (0.8%)	47 (39.8%)	47 (39.8%)	23 (19.5%)	<0.001	0.985
Self-doubt	Before	39 (33.1%)	66 (55.9%)	11 (9.3%)	2 (1.7%)		
	During	8 (6.8%)	64 (54.2%)	38 (32.2%)	8 (6.8%)	<0.001	0.949
Physical exercise	Before	7 (5.9%)	39 (33.1%)	31 (26.3%)	41 (34.7%)		
	During	24 (20.3%)	53 (44.9%)	21 (17.8%)	20 (16.9%)	<0.001	0.896

**Figure 4:**
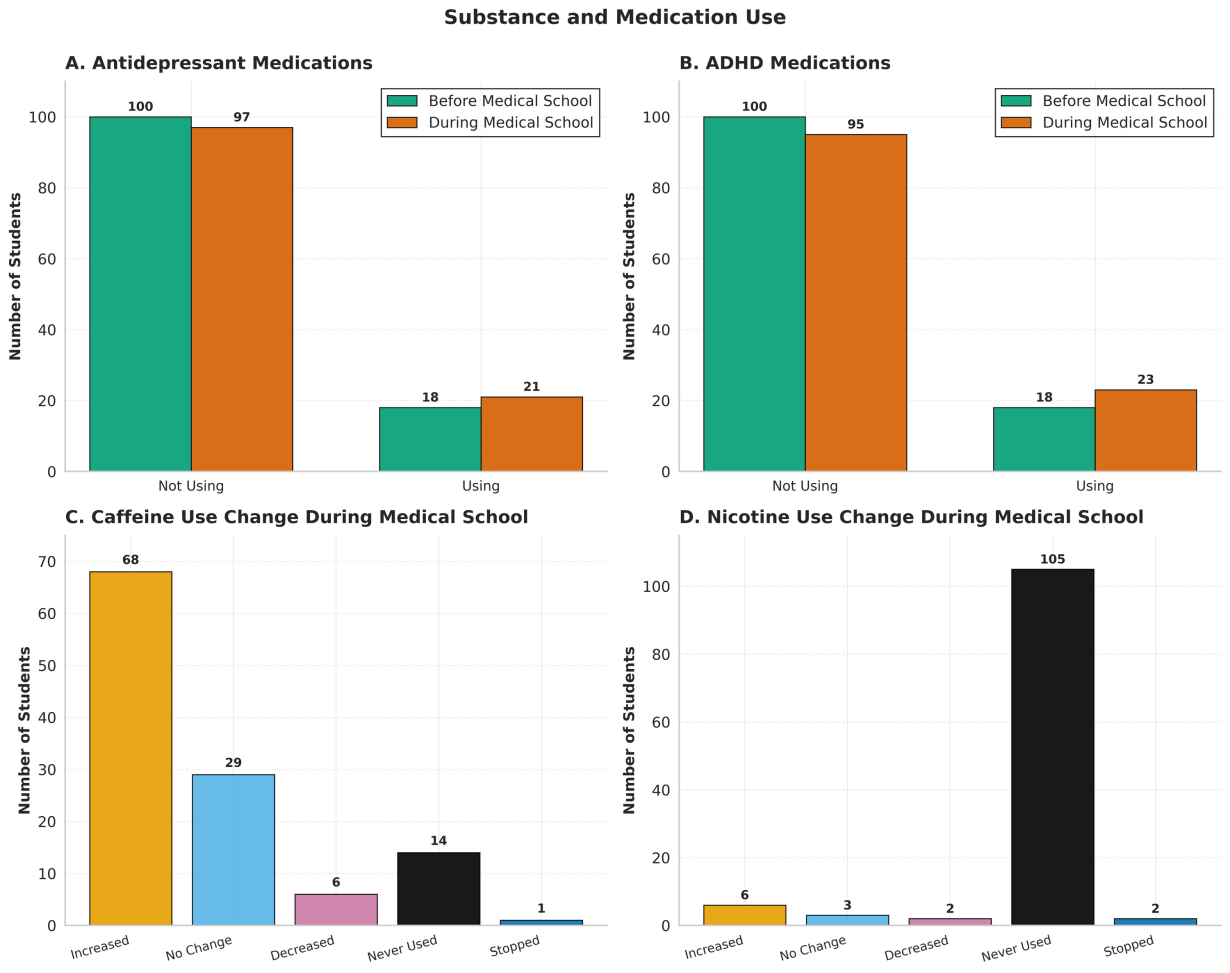
Substance and medication use. No statistically significant relationships were identified according to McNemar’s test. ADHD, attention-deficit/hyperactivity disorder

Participants were asked about their use of antidepressants, attention-deficit/hyperactivity disorder (ADHD) medication, caffeine use, and nicotine use (Figure 4). Overall use of antidepressant medications stayed roughly constant before and after starting medical school (Figure 4A). The same can be said for ADHD medications (Figure 4B). A total of 13 (11.0%) students reported starting antidepressants, and 10 (8.5%) students reported stopping for a net change of +3 (2.5%) students. For ADHD medications, 13 (11.0%) students started the medications while 8 (6.8%) discontinued for a net change of +5 (4.2%) students (Table 5). Neither of the small changes reached statistical significance using McNemar’s test (p=0.68 for antidepressant change and p=0.38 for ADHD medication change). Caffeine use was popular among survey participants, with 103 (87.3%) reporting using the substance and 68 (57.6%) increasing their use (Figure 4C). Conversely, 105 (89%) of students reported never using nicotine (Figure 4D). 

**Table 5:** Changes in psychiatric medication use during medical school. Data represent raw frequency counts (*n*) for a total of 118 participants and percentages of the total. Statistical significance for paired categorical shifts was determined using McNemar's test.

Medication type	Never used	Started during	Stopped during	Continued throughout	Net change	*P*-value
Antidepressants	87 (73.7%)	13 (11.0%)	10 (8.5%)	8 (6.8%)	+3 (2.5%)	0.68
ADHD medications	87 (73.7%)	13 (11.0%)	8 (6.8%)	10 (8.5%)	+5 (4.2%)	0.38

**Figure 5:**
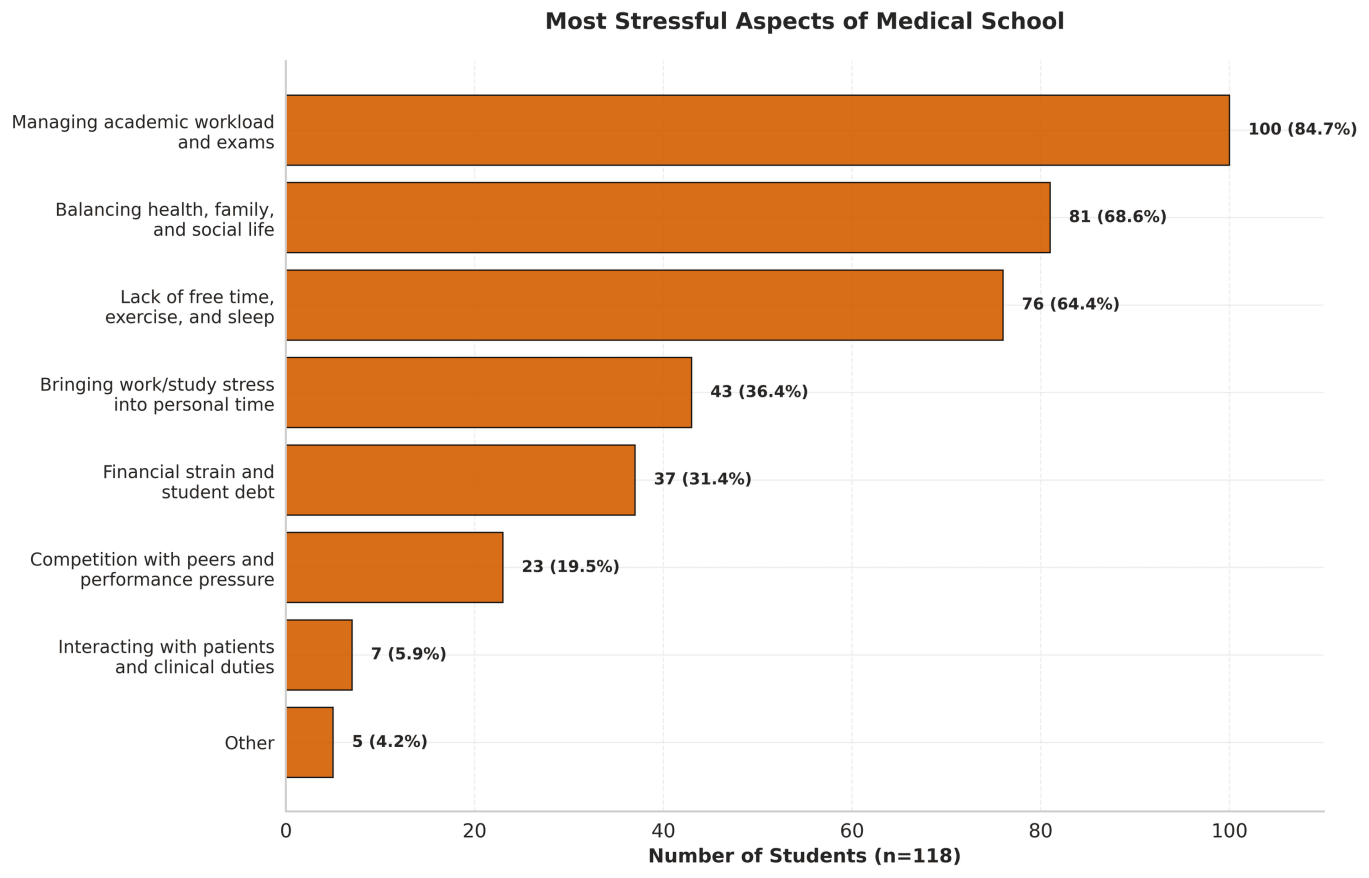
Most stressful aspects of medical school. Participants' answers to the question: In your opinion, what is the most stressful aspect of medical school? (Select three choices.)

Participants were asked to select three items from a list of stressors to determine the most common stressors among medical students (Figure 5).

## Discussion

The demographic profile of our sample reflects broader national trends in medical education. The slight female majority in our cohort (67, 56.8%) aligns closely with the national cohort of students who matriculated into osteopathic medical schools at 56.2% [13]. A representative balance of genders is important as females tend to report higher levels of stress, burnout, and depressive symptoms as compared to their male counterparts [14-16]. Another important demographic consideration to keep in mind is that 110 (93.2%) of our respondents were in their first three years of medical school. It has been reported that the prevalence of mental distress is different depending on the year of study, although this finding is inconsistent and may be institution-dependent [1,2,17].

Our finding of a large increase in self-doubt is consistent with other studies related to this concept. 46 (38.9%) of our survey respondents reported feelings of self-doubt more than half the days or nearly every day. Liu et al. found that 49% of the first-year students they surveyed reported a moderate to high amount of self-doubt [18]. Self-doubt is closely related to impostor phenomenon, defined as a persistent inability to internalize success despite objective evidence of high achievement [19]. Screening tools like the Young Imposter Scale have identified this phenomenon in 40% of medical students [19]. Recognizing the high prevalence of impostor phenomenon may lead to targeted interventions early on in medical school. Since imposter phenomenon is associated with other types of emotional distress, addressing these feelings of inadequacy might help prevent distress and burnout in future physicians [20].

The decline in physical activity observed in our cohort is a particularly concerning finding given the well-established bidirectional relationship between exercise and mental health. The proportion of students reporting no regular exercise increased 3.4-fold during medical school, and those exercising frequently more than half the days fell by 31 (26.3%). Prior research has demonstrated that emotional exhaustion is inversely correlated with exercise in medical students, and that physical activity is associated with reduced burnout and improved quality of life [21]. The loss of exercise as a coping resource may therefore compound the psychological burden that medical training imposes, creating a negative feedback loop in which worsening mental health reduces motivation to exercise, which in turn worsens mental health.

Perhaps the most clinically substantial finding of this study is the disconnect between worsening mental health symptoms and stable psychiatric medication use. Use of prescribed antidepressants (*P *= 0.68) and ADHD medications (*P *= 0.38) did not significantly change between the pre-medical school and during-medical school periods, despite substantial increases in reported exhaustion, self-doubt, and stress. Several reasons might explain this contradiction. It has previously been reported that among medical students who screen positive for depression, only 15.7% will seek medical attention [4]. Stigma might be dissuading students from getting necessary care [22]. It is also possible that the increase in mental distress does not rise to the level of a mental disorder that is commonly treated with medication. Finally, students might be underreporting their current use of psychiatric medication.

This study has several limitations. The cross-sectional design and retrospective self-report of pre-medical school mental health introduce the possibility of recall bias; students experiencing significant current distress may systematically overestimate how well they felt before medical school, potentially inflating the observed within-subject differences. Furthermore, the custom survey instrument was not formally pilot-tested or evaluated for psychometric validity. Participants were drawn from a single osteopathic medical school, which limits the generalizability of findings to other institutions. Additionally, the relatively small sample size of 118 participants may limit the statistical power to detect more subtle shifts in medication use or substance behaviors. Despite these constraints, the study’s strengths include a representative gender balance, coverage of the first three years of medical school, and a novel focus on the within-individual change in health behaviors and psychiatric medication.

A central challenge in medical education is distinguishing between maladaptive distress and the productive pressure required for professional growth. While the observed increases in self-doubt and exhaustion are concerning, it remains an open question whether a certain threshold of these experiences is necessary for students to push themselves toward clinical excellence. However, the decrease in exercise is likely to be harmful. Institutional shifts like pass/fail grading may improve well-being, though their impact on academic incentives and clinical excellence remains debated [23]. Striking a balance that fosters resilience without causing psychological damage is critical for medical curriculum design.

Future research may benefit from the use of prospective methodologies to remove the recall bias that is inherent in retrospective self-reporting. A critical area for further investigation is the identified discordance between escalating psychological distress and static pharmacological utilization. It remains unclear whether the observed increase in symptoms fails to meet clinical diagnostic thresholds or if structural barriers and pervasive stigma effectively preclude formal treatment. Additionally, correlating psychological stress with objective academic performance could help identify a healthy medium where professional challenge fosters growth without inducing psychological harm. Finally, evaluating the direct impact of specific curricular interventions on student well-being will be essential for developing a sustainable training environment.

## Conclusions

This study adds to a growing evidence base demonstrating that medical school is associated with meaningful worsening of mental health symptoms and health-promoting behaviors and that this deterioration is not being adequately addressed through formal psychiatric treatment. The findings point toward a need for proactive, institution-level interventions that address structural contributors to student distress: reducing unnecessary academic burden, protecting time for physical activity and social connection, creating destigmatized pathways to mental health support, and building cultures in which self-doubt and psychological difficulty can be acknowledged openly. Future prospective studies with objective measures and longitudinal designs will be important for disentangling the causal mechanisms underlying these patterns and evaluating the effectiveness of targeted interventions.

## Appendices

Appendix

**Table 6:** Full Survey Instrument This questionnaire assesses mental health status, health-promoting behaviors, and medication use prior to and during medical school. The demographic, lifestyle, and medication use items are the authors' original creation (Credits: Amira Katrib, Asis Babun, Kelson Knighton, Thomas Bigham). The psychological distress items are adapted from the Patient Health Questionnaire-9 (PHQ-9) [10], the General Anxiety Disorder-7 (GAD-7) [11], and the Social Functioning Scale (SFS) [12]. The original questionnaires were substantially modified as allowed by their original licenses. This novel questionnaire is published under a Creative Commons Attribution 4.0 International (CC BY 4.0) License.

#	Survey Question	Response Options
1	What is your age group?	18-24; 25-29; 30-34; 35+
2	Gender	Male; Female; Non-binary / third gender; Prefer not to say
3	Year of Medical School	I; II; III; IV; Graduate
4	Before continuing this survey, please assess how you are currently feeling mentally?	Not feeling well at all.; Feeling neither good nor bad.; Feeling good.; Feeling great.
5	Were you prescribed any of the following BEFORE Medical School? SSRI/SNRI/TCA/MAO-I/Atypical Anti-depressant/Anti-psychotics?	YES; NO
6	Were you prescribed any of the following DURING Medical School? SSRI/SNRI/TCA/MAO-I/Atypical Anti-depressant/Anti-psychotics?	YES; NO
7	SINCE STARTING medical school, how has your usage/dosage of SSRI/SNRI/TCA/MAOI/atypical antidepressant/anti-psychotic changed?	Increased; Decreased; Remained the same; No longer taking it; Never taken
8	BEFORE entering medical school, were you ever prescribed an ADHD/ADD medication (e.g., Adderall, Ritalin, Vyvanse, etc.)?	YES; NO
9	Have you been prescribed an ADHD/ADD medication DURING medical school?	YES; NO
10	SINCE STARTING medical school, how has your usage/dosage of ADHD/ADD medications changed?	Increased; Decreased; Remained the same; No longer taking it; Never taken
11	BEFORE medical school: On average, how much caffeine (coffee, energy drinks, tea, caffeine pills, etc.) did you consume per day?	None at all; Less than 100 mg (about 1 small cup of coffee); 100-200 mg (about 1-2 cups); 201-400 mg (about 3-4 cups); More than 400 mg (more than 4 cups)
12	DURING medical school: How has your usage of caffeine changed since starting medical school?	Increased; Decreased; Remained the same; No longer taking it; Never taken
13	BEFORE medical school: Did you use nicotine products (cigarettes, vaping, nicotine gum, etc.)?	None at all; Less than 5 mg/day (e.g., 1-2 cigarettes or equivalent in vaping/nicotine gum); 5-10 mg/day (e.g., 3-5 cigarettes or equivalent); 11-20 mg/day (e.g., 1 pack of cigarettes or equivalent); More than 20 mg/day (e.g., more than 1 pack of cigarettes or equivalent)
14	DURING medical school: How has your usage of nicotine changed since starting medical school?	Increased; Decreased; Remained the same; No longer taking it; Never taken
15	BEFORE medical school, how often would you have felt physically or mentally drained?	Not at all; Several days; More than half the days; Nearly every day
16	DURING medical school, how often would you have felt physically or mentally drained?	Not at all; Several days; More than half the days; Nearly every day
17	BEFORE medical school, how often have you experienced self-doubt or feelings of letting others down?	Not at all; Several days; More than half the days; Nearly every day
18	DURING medical school, how often have you experienced self-doubt or feelings of letting others down?	Not at all; Several days; More than half the days; Nearly every day
19	In your opinion, what is the most stressful aspect of medical school? (select 3 choices)	Managing academic workload and exams; Financial strain and student debt; Balancing health, social life, and family responsibilities; Competition with peers and performance pressure; Interacting with patients and clinical duties; Bringing work and study stress into personal time; Lack of free time, exercise, and sleep; Other
20	BEFORE medical school, how often did you partake in any mindfulness exercises such as yoga and meditation?	Not at all; Several days; More than half the days; Nearly every day
21	DURING medical school, how often do you partake in any mindfulness exercises such as yoga and meditation?	Not at all; Several days; More than half the days; Nearly every day
22	BEFORE medical school, how often did you partake in physical exercise such as gym-going, hiking, or biking?	Not at all; Several days; More than half the days; Nearly every day
23	DURING medical school, how often do you partake in physical exercise such as gym-going, hiking, or biking?	Not at all; Several days; More than half the days; Nearly every day
24	How often have you felt overwhelmed by responsibilities or daily tasks?	Never; Occasionally; More than half the time; Almost every day
25	Have you been feeling more tense, restless, or on edge than usual?	Not at all; Sometimes; Often; Almost always
26	How frequently have you had trouble relaxing or calming your thoughts?	Never; Occasionally; More than half the time; Almost every day
27	Have you found it difficult to focus on tasks or conversations due to stress or anxiety?	Never; Occasionally; More than half the time; Almost every day
28	Have you experienced physical symptoms of stress, such as headaches, muscle tension, or stomach discomfort?	Not at all; Sometimes; Often; Almost always
29	How often do you reach out to friends, family, or acquaintances for social interaction?	Daily; A few times per week; Occasionally; Rarely or never
30	How comfortable do you feel engaging in conversations or group activities?	Extremely uncomfortable; Moderately uncomfortable; Slightly uncomfortable; Neither comfortable nor uncomfortable; Slightly comfortable; Moderately comfortable; Extremely comfortable
31	Do you feel that stress or anxiety has negatively impacted your ability to maintain social relationships?	Extremely negative; Moderately negative; Slightly negative; Neither positive nor negative; Slightly positive; Moderately positive; Extremely positive
32	How often have you avoided social situations due to feeling overwhelmed, anxious, or exhausted?	Never; Occasionally; More than half the time; Almost every day
33	Have you been able to participate in hobbies, events, or group activities that you enjoy?	Yes, regularly; Occasionally; Rarely; Not at all
34	After completing this survey, please assess how you are currently feeling mentally.	Not feeling well at all.; Feeling neither good nor bad.; Feeling good.; Feeling great.
